# *PDE4* Gene Family Variants Are Associated with Response to Apremilast Treatment in Psoriasis

**DOI:** 10.3390/genes15030369

**Published:** 2024-03-17

**Authors:** Kalliopi Liadaki, Efterpi Zafiriou, Themistoklis Giannoulis, Sofia Alexouda, Kleoniki Chaidaki, Polyxeni Gidarokosta, Angeliki-Viktoria Roussaki-Schulze, Sotirios G. Tsiogkas, Athina Daponte, Zissis Mamuris, Dimitrios P. Bogdanos, Nicholas K. Moschonas, Theologia Sarafidou

**Affiliations:** 1 Department of Biochemistry and Biotechnology, University of Thessaly, Viopolis, 41500 Larissa, Greece; kliad@uth.gr (K.L.); zmamur@uth.gr (Z.M.); 2 Department of Dermatology, Faculty of Medicine, School of Health Sciences, University of Thessaly, Viopolis, 41500 Larissa, Greece; zafevi@med.uth.gr (E.Z.); kleonikichaidaki@gmail.com (K.C.); xeniagid@gmail.com (P.G.); roussaki@otenet.gr (A.-V.R.-S.); 3 Department of Animal Science, University of Thessaly, Gaiopolis, 41334 Larissa, Greece; themisgia@gmail.com; 4 Department of Rheumatology and Clinical Immunology, Faculty of Medicine, School of Health Sciences, University of Thessaly, Viopolis, 41500 Larissa, Greece; stsiogkas@med.uth.gr (S.G.T.); athena.dap23@gmail.com (A.D.); bogdanos@uth.gr (D.P.B.); 5 School of Medicine, University of Patras, 26500 Patras, Greece; 6 Foundation for Research and Technology Hellas, Institute of Chemical Engineering Sciences, 26504 Patras, Greece

**Keywords:** apremilast, psoriasis, association study, SNP, PDE4, CYP3A4, protein interactome, pathway analysis

## Abstract

Moderate-to-severe psoriasis (Ps) treatment includes systemic drugs and biological agents. Apremilast, a small molecule primarily metabolized by cytochrome CYP3A4, modulates the immune system by specifically inhibiting phosphodiesterase type 4 (PDE4) isoforms and is currently used for the treatment of Ps and psoriatic arthritis (PsA). Clinical trials and real-world data showed variable efficacy in response among Ps patients underlying the need for personalized therapy. This study implements a candidate-gene and a network-based approach to identify genetic markers associated with apremilast response in forty-nine Greek Ps patients. Our data revealed an association of sixty-four SNPs within or near *PDE4* and *CYP3A4* genes, four SNPs in ncRNAs *ANRIL*, *LINC00941* and *miR4706*, which influence the abundance or function of PDE4s, and thirty-three SNPs within fourteen genes whose protein products either interact directly with PDE4 proteins or constitute components of the cAMP signaling pathway which is modulated by PDE4s. Notably, fifty-six of the aforementioned SNPs constitute eQTLs for the respective genes in relevant to psoriasis tissues/cells implying that these variants could be causal. Our analysis provides a number of novel genetic variants that, upon validation in larger cohorts, could be utilized as predictive markers regarding the response of Ps patients to apremilast treatment.

## 1. Introduction

Psoriasis (Ps) is a chronic autoimmune inflammatory disease with a multifactorial etiology and a prevalence estimate of 2–3% worldwide [1]. The management of moderate-to-severe psoriasis involves various treatment approaches, i.e., first-line systemic drugs, small molecules, and biological agents [2]. Although these treatments are generally effective, they show considerable variation in response rates among patients [3]. It is estimated that 30–50% of the patients present an insufficient response to various treatments, which depends to a significant degree on the genetic background [4]. Furthermore, an appreciable number of patients, who exhibit an initial response to treatment, experience a secondary loss of response, while others discontinue treatment due to side effects [5,6]. Hence, the identification and implementation of valid prognostic biomarkers in the context of precision medicine are of high importance for the protection of patients’ quality of life as well as for cost reduction in healthcare.

Apremilast, a small molecule which specifically inhibits the 3′,5′ cyclic adenosine monophosphate (cAMP) specific phosphodiesterase type 4 (PDE4) and modulates the immune system, has been added to the armamentarium for the efficacious treatment of moderate-to-severe plaque Ps, psoriatic arthritis (PsA) and oral ulcers of Behcet’s disease in adults after FDA approval in 2014 [7]. Due to its low molecular weight, apremilast is easily absorbed from the gut and transferred into the cells where it hinders PDE4 [8]. PDE4, a major subtype of PDEs expressed by immune or inflammatory cells, is a well conserved enzyme encoded by four genes, *PDE4A*, *B*, *C* and *D* mapped to chromosomes 19p13.2, 1p31.3, 19p13.11, and 5p11.2-q12.1, respectively. Due to extensive alternative splicing and/or the use of different promoters, they generate multiple alternatively spliced transcripts producing many different functional isoforms [9]. It has been shown by in vitro experiments that apremilast specifically inhibits the PDE4B1, PDE4C1, PDE4B2, PDE4D2 and PDE4A1A isoforms without targeting any cell surface receptors and kinases [10]. Inhibition of PDE4s results in an increase in the intracellular second messenger cAMP as its hydrolysis to 5′-AMP is performed exclusively by these enzymes. Consequently, various signaling pathways are modified in immune cells, resulting in the decrease in proinflammatory cytokines and the increase in anti-inflammatory mediators [11]. Apremilast is primarily metabolized by cytochrome 450 (CYP-450) 3A4, whose expression and activity in the liver and small intestine is highly variable in part due to genetic factors [12].

Although apremilast has advantages, i.e., oral administration, lack of immunogenicity, acceptable levels of safety and tolerance, and relatively low cost, different response rates have been reported. Earlier phase III trials have shown that after 16 weeks of treatment 29–41% of the patients showed at least a 75% reduction in the Psoriasis Area and Severity Index (PASI) score [13,14,15]. Accordingly, subsequent real-world data showed variable efficacy, underlying the need for personalized therapy [16,17,18]. In this context, there is only one published pharmacogenomics study examining the association between SNPs and apremilast response outcome concerning 34 Russian patients with Ps [19]. The authors performed a genome-wide association study (GWAS) in combination with an analysis of pre-selected SNPs, based on their association with Ps or PsA, and identified SNPs associated with apremilast response which map in four genomic regions of unknown function and in *IL-1β*, *IL-4*, *IL-23R*, and *TNF-α* genes. 

Typical pharmacogenetic analyses aiming to determine genetic markers for drug response primarily focus on polymorphisms on either direct drug targets or drug metabolizing enzymes. In this aspect, we performed an association study on polymorphisms inside or adjacent to *PDE4A*, *B*, *C*, *D* and *CYP3A4* and extend the analysis to ncRNAs and proteins that may affect PDE4 function and therefore the response to apremilast treatment, in a cohort of 49 Greek patients.

## 2. Materials and Methods

### 2.1. Patients 

Forty-nine adult patients diagnosed with Ps vulgaris who had been under treatment with apremilast (trade name Otezla, 30 mg taken orally twice/day) for at least 6 months were included in the study. The patients were recruited and followed-up at the Dermatology Department of the General University Hospital of Larissa (GUHL), Greece. Patients’ response was evaluated based on the decrease in the PASI score at 6 and 9 months of treatment and a reduction greater than 75% classified them as responders. Responder patients receiving apremilast in combination with another treatment as well as patients who exhibited a marginal positive or negative response to apremilast were excluded from the study. This study was conducted after informed consent of the patients with the approval of the Research Ethics Committee of GUHL (11/2nd/08-02-2022) in accordance with the Declaration of Helsinki. Patients’ peripheral blood and DNA samples were semi-anonymized with a code number and treated as confidential. 

### 2.2. Samples, Genotyping, Quality Control and Association Analysis

DNA extraction was performed from peripheral whole blood using the PureLink Genomic DNA Kit (Thermo Fisher Scientific, Waltham, MA, USA) according to the manufacturer’s instructions. DNA integrity/quality was assessed through agarose gel electrophoresis and the quantification was performed with a Quawell spectrophotometer. DNA concentration was adjusted to 50 ng/μL. Genotype screening was performed at the Human Genomics Facility (HuGe-F) of Erasmus MC (University Medical Centre, Rotterdam, The Netherlands) using 200 ng DNA per sample and the Illumina Infinium Global Screening Array MD v3.0 which contains approximately 730,000 SNP variants. 

The genotyping data were obtained in .ped and .map files. Quality control steps were applied that filtered out individuals and SNPs with call rate < 99%, SNPs with minor allele frequency (MAF) < 5% and linkage disequilibrium (LD) > 0.8 based on r^2^ values. The genomic coordinates for all the genes tested for association with apremilast response were retrieved from the Ensembl database (https://www.ensembl.org/index.html accessed on 15 December 2023), ref. [20] using the GRCh37 assembly in which all the genotyping data derived from HuGe-F were available. Based on these coordinates, an association analysis for allelic, dominant, recessive and genotypic models was implemented. The odds ratio was calculated with 95% confidence interval based on the comparative risk of non-response in the presence of the minor allele. All the above analyses were performed using the PLINK software (PLINK 1.9) [21]. Furthermore, the clinical characteristics of the patients were assessed for association with the response to apremilast applying the appropriate statistics using R Studio.

### 2.3. ncRNA Analysis

The LncRNA2Target v3 database [22], which contains exclusively experimentally validated data, was used to retrieve all the human lncRNAs/mRNA pairs and the respective Ensembl IDs. SNPs located within these lncRNAs were tested for association with the treatment response as described in Section 2.2. 

Τhe miRTarBase [23], which includes only experimentally verified miRNA-mRNA interactions, was searched to track down all the miRNAs that target *PDE4* or *CYP3A4* mRNAs as well as the miRNA-related SNPs. The miRNA identifiers were used as queries in the Ensembl database and those that did not correspond to a unique gene or were discontinued were excluded from further analysis. Furthermore, miRNA-related variants that did not map to a unique genomic location according to the Ensembl database were also excluded. The remaining miRNA-related SNPs were investigated for LD data in all available populations using the Linkage Disequilibrium Calculator tool provided by the Ensembl database, setting the window size at 0.5 Mb and the threshold for r^2^ and D’ at 0.7. The list of SNPs that either map within the miRNAs or display high LD with them were examined for association with the response to apremilast as described in Section 2.2. 

### 2.4. Protein-Network and Pathway Analysis 

Protein–protein interaction (PPI) data for human PDE4 proteins were extracted from the PICKLE meta-database (version 3.3) [24,25], using the “first neighbors” network setup and the “cross-checking” filtering method which includes only the experimentally validated with high confidence direct PPI output. The Cytoscape software platform ([26], version 3.8.2, http://www.cytoscape.org/ accessed on 10 January 2024) was used for the reconstruction of the protein interactome. The Kyoto Encyclopedia of Genes and Genomes (KEGG) database was used to retrieve the cAMP signaling pathway (hsa04024) which is regulated by PDE4 proteins. We created a simplified version of the pathway focusing on the signal transduction relevant to inflammation psoriasis. The SNPs within the genes encoding the proteins of the PPI network and the modified KEGG pathway were tested for association with the response to apremilast as described in Section 2.2.

### 2.5. Expression Quantitative Trait Loci (eQTL) Analysis

The data for the eQTL analysis were derived from the Ensembl database by setting > 1.3 as the threshold of the *p* value (−log10). The *p* values were converted to non-log values. From the list of tissue/cells in which the variant of interest was found to be correlated with the respective gene expression, only the skin, blood and immune cells were selected for all the genes except for *CYP3A4*, for which the liver and small intestine were selected. The effect size of the eQTLs refers to the effect of the alternative versus the reference allele. 

## 3. Results

### 3.1. Clinical Characteristics of the Study Population

Forty-nine patients, 32 males and 17 females, with Ps vulgaris who were treated with apremilast were enrolled in this study, and their clinical characteristics were analyzed. Their mean age was 55.4, and the mean disease duration was 18.4 years. At baseline, the mean PASI was 10.3, the mean BMI was 31.6, and the mean weight was 92.8 kg. In addition to BMI, the baseline weight was also included in the analysis due to the body-weight-independent treatment dosing of apremilast. Comorbidities were reported in approximately 70% of the patients. In accordance with real-world data, the proportion of the patients that achieved PASI score improvement greater than 75% after 24–36 weeks of treatment was 45% [16,17,18]. Thus, the responder and non-responder groups include 22 and 27 patients, respectively. Table 1 shows the main characteristics of the study population. Their sex, age, disease duration, baseline BMI and weight are not correlated with the efficacy of apremilast. However, a higher absolute baseline PASI is associated with lower treatment response. 

### 3.2. PDE4 and CYP3A4 Variants Are Associated with Response to Treatment

Following the genotyping data quality control, 3892 SNPs which are mapped within a physical distance of approximately ±1 Mb of PDE4A, PDE4B, PDE4C, PDE4D and CYP3A4 genes’ chromosomal location were selected. A subsequent filter removed 2628 SNPs with MAF < 5% and pruned for LD > 0.8 based on r^2^ values which further excluded 280 SNPs. Thus, for the association analysis regarding apremilast response, 984 SNPs were finally employed. Allele-based association tests detected 64 SNPs displaying nominal *p* values < 0.05 (Table 2) including 10 SNPs with *p* values ≤ 0.01. Apart from the allelic association, SNP rs1045895 also shows association with the response under the dominant model of inheritance (Table 2). Interestingly, 15 SNPs associated with the apremilast response map within intronic regions of PDE4B, PDE4D and CYP3A4.

### 3.3. SNPs within ncRNAs That Target PDE4 mRNAs Are Associated with Apremilast Response

Differential response to apremilast treatment may be affected by various biological factors, including lncRNAs and miRNAs that target *PDE4* and *CYP3A4* genes influencing their stability, level of abundance or function. Therefore, all human lncRNAs/mRNA pairs were retrieved from LncRNA2Target v3 database, and SNPs located within the lncRNA genes were examined for association with response to apremilast. In total, 18 lncRNAs were correlated with PDE4A, B and C expression levels. Interestingly, two lncRNAs, i.e., *ANRIL* and *LINC00941*, were identified to contain SNPs that are associated with the response to apremilast (Table 3). The genomic coordinates of lncRNAs, their SNP content for which genotyping data are available in our analysis and the corresponding *PDE4* gene(s) are presented in Table S1. 

Similarly to lncRNAs, SNPs which could potentially affect miRNA-mRNA pairing may result in modified transcription or translation of their target transcripts. Τhe miRTarBase lists 201 entries for miRNAs which contain 1276 SNPs (Table S2) and target *PDE4* or *CYP3A4* transcripts. However, only one of those SNPs is included in the genotyping platform used in our analysis. Thus, we tracked down all the SNPs that exhibit high linkage disequilibrium values (r^2^ > 0.7) with the miRNAs-related SNPs and examined for those having available genotypic data. In total, 3305 SNPs were identified with 15 of them been genotyped in our analysis. Out of these, rs2296316 (A/G) shows a positive association between the presence of the alternative allele (A) and the response to the apremilast therapy (Table 3). The aforementioned SNP is in complete LD (r2 = 1, D’ = 1) with rs2296319 in miR4706 which targets *PDE4C* mRNA.

### 3.4. Protein Network and Pathway Analysis

**Figure 1:**
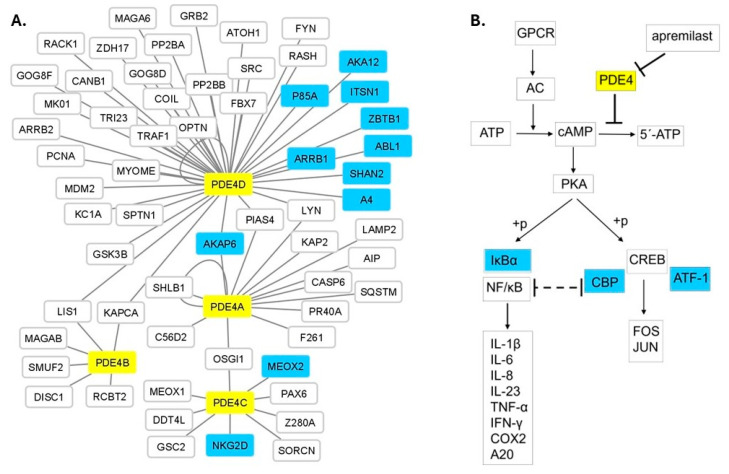
(**A**) The protein interactome of PDE4A, B, C and D with their interconnecting protein neighbors. PDE4A, B, C and D (yellow nodes) are interconnected with six proteins (AKAP6, PIAS4, LYN, KAPCA, LIS1, OSGI1). PDE4D is the most central node, whereas PDE4B is the most peripheral PDE4 protein of the network. SNPs located within eleven PDE4-first neighbor protein (light blue nodes) coding genes showed statistically significant association with the response to apremilast treatment (Table 4). Forty-six additional protein nodes (white nodes) constitute the entire PDE4 protein network. (**B**) Part of the cAMP signaling pathway showing apremilast’s mechanism of action. SNPs within *IκΒα*, *CBP* and *ATF-1* (light blue colored) coding genes are associated with apremilast treatment response (Table 4).

Τhe response to a drug treatment is generally the result of several functionally interconnected biological factors. Consequently, it is anticipated that a network-based approach may be a reliable source for the identification of predictive biomarkers in addition to those determined by the candidate gene approach, regarding the response to apremilast treatment. Accordingly, we reconstructed the interactome of PDE4A, B, C and D including their first protein neighbors (Figure 1). The network consists of 65 protein-nodes with 69 interactions. PDE4A, B, C and D are interconnected with six proteins. Namely, PDE4D, the most central node of the network having 39 first protein neighbors, is connected with both PDE4A and PDE4B, by three (AKAP6, LYN, PIAS4) and two (LIS1 and KAPCA) proteins, respectively, whereas PDE4A is connected with PDE4C by one protein (OSGI1). The association analysis of the response to apremilast treatment with the SNPs located within the genes encoding the protein nodes of the network detected 33 SNPs in 11 genes with statistically significant *p* values (Table 4). The respective proteins are mainly first interactors of the PDE4D protein (Figure 1A). Along the same lines, a simplified version of the cAMP-signaling KEGG pathway focusing on the signal transduction relevant to psoriasis was created (Figure 1B), and its analysis revealed SNPs within *CBP*, *ATF1* and *NFKBIA* genes to be associated with the response to apremilast.

### 3.5. eQTL Analysis

**Figure 2:**
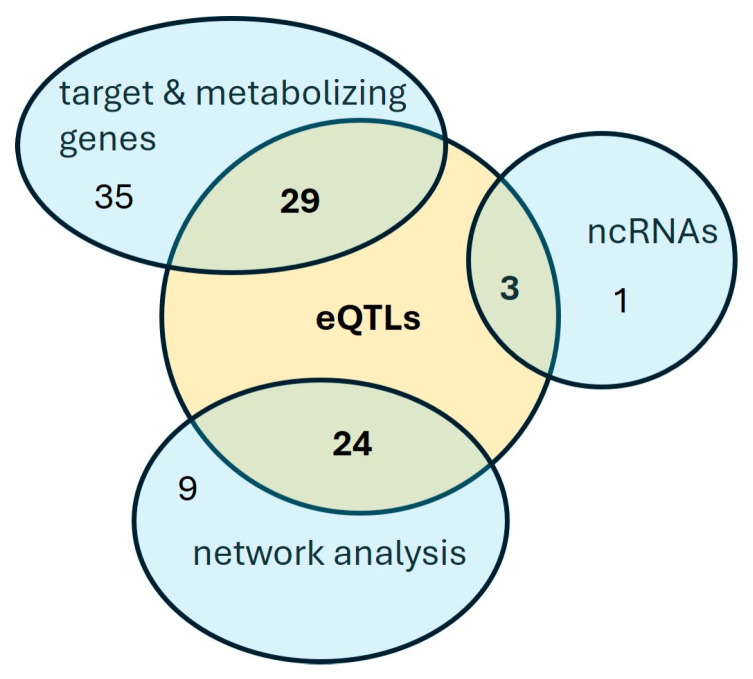
A Venn diagram of the SNPs associated with the apremilast response.

The majority of the SNPs associated with the response to apremilast (Table 2, Table 3 and Table 4) are mapped either in intronic regions or in the chromosomal vicinity of the respective genes. Thus, they may contribute to the regulation of the corresponding gene expression or may be in high LD with other regulatory variants not included in the genotyping platform used in our analysis. In order to identify putative causal variants, we examined whether these SNPs constitute significant cis-eQTLs in tissues or cells relevant to the examined phenotype. In total, 56 eQTLs were determined corresponding to 55,4% of the total SNPs identified with the complementary approaches described previously (Figure 2). Table S3 lists the eQTLs whose alternative alleles display an effect size ranging from 0.04 to 1.95 and the tissues in which the correlation with the expression of the respective gene was identified. The majority of the eQTLs were identified in immune cells, whereas twenty-two were also detected in the skin. Two SNPs located within and upstream of the CYP3A4 gene constitute eQTLs in the liver and the small intestine, respectively.

## 4. Discussion

In this pharmacogenetic study, we have implemented a candidate-gene approach at three different levels, i.e., (i) the apremilast direct target and its metabolizing enzyme genes, (ii) the ncRNAs that are associated or influence their expression and (iii) the PDE4 protein network and pathway analysis gene products. The genotypic data of the 49 patients propose an association with the response to apremilast of (i) sixty-four SNPs within or near *PDE4* and *CYP3A4* genes, (ii) three SNPs within the *ANRIL* and *LINC00941* lncRNAs and one SNP inside *miR4706* and (iii) thirty-three SNPs within genes encoding protein components of the PDE4 interactome or cAMP signaling pathway relevant to psoriasis.

In order to evaluate the identified associations (listed in Table 2, Table 3 and Table 4) and their potential clinical application, we explored the literature for previous associations with autoimmune diseases or diseases with an autoimmune component. Indeed, previous studies have shown that SNPs associated with the response to Ps treatment are also associated with Ps susceptibility [27,28]. Specifically, rs892085 and rs322151, located downstream of *PDE4A*, have been associated with Ps and PsA [29,30,31] and systemic sclerosis, respectively [32]. In addition, rs2395022, mapped 0.6 Mb downstream of *CYP3A4* constitutes a susceptibility locus for inflammatory bowel disease [33]. Furthermore, rs2305795 and rs11085752, upstream and downstream of *PDE4A*, respectively, have been shown to contribute to the genetic risk of neurological diseases, i.e., narcolepsy and Alzheimer’s [34,35]. The rs2239316 of *CBP* gene is associated with *IL32* CpG methylation in CD4+ and CD8+ T cells, which in turn is associated with juvenile idiopathic arthritis [36]. Finally, rs696, located in the 3′ UTR of *NFKBIA* gene, was associated with Guillain–Barré syndrome [37], Behcet disease [38] and anti-TNF response in patients with Crohn’s disease and ulcerative colitis [39].

The analysis of lncRNAs showed that SNPs within *ANRIL* and *LINC00941*, which are correlated with their expression in immune cells and skin (eQTLs listed in Table S3), are also associated with the response to apremilast (Table 3). The association with *ANRIL* lncRNA is of particular importance since *ANRIL* has been previously associated with autoimmune disease susceptibility, including Ps [40,41,42]. Previous studies in T-Rex 293 HEK cell lines or epidermal tissue cultures have demonstrated that following *ANRIL* or *LINC00941* knockdown, *PDE4B* expression was down or up regulated, respectively [22,43]. Although this correlation could be indirect and may be detected due to the sequestration of specific miRNAs, it provides evidence for the putative cause of the observed genetic association. This, in combination with the findings of the involvement of *LINC00941* in human epidermal homeostasis [44] as well as the regulation of TGF-β/Smad signaling by ANRIL [45], also suggests the molecular mechanisms that are linked with the response.

The applied protein network analysis for the detection of putative biomarkers identified SNPs in 11 genes associated with apremilast treatment response (Figure 1A), four of which have established roles related to PDE4s function. Specifically, AKAP6, a common first neighbor of PDE4A and PDE4D, belongs to the family of A-kinase anchoring proteins (AKAPs) and, among other functions, is involved in cAMP-mediated signaling, forming a complex which includes protein kinase A (PKA), Rap guanine nucleotide exchange factor 3 (PRGF3/EPAC1) and different phosphodiesterases including PDE4D3 [46,47]. AKA12, also an AKAP family member, functions as an anchoring protein mediating the subcellular compartmentation of PKA [48]. ARRB1 is involved in the degradation of cAMP by recruiting cAMP phosphodiesterases to ligand-activated receptors [49] and ZBTB1 is a transcription factor that represses cAMP-responsive element (CRE)-mediated transcriptional activation [50]. KEGG pathway analysis identified three genes (Figure 1B) with pivotal roles in the cAMP signaling pathway, i.e., *CBP*, *ATF1* and *NFKBIA*. The CBP protein, through specific binding to phosphorylated CREB, enhances its transcriptional activity towards cAMP-responsive genes [51]. ATF1 is activated by PKA phosphorylation and stimulates CRE-containing gene expression [52]. NFKBIA inhibits the activity of NF-kappa-B, modulating immune and pro-inflammatory responses [53]. The aforementioned functional roles provide meaningful information for the detected genetic associations. On the other hand, the rest of the associated genes (Figure 1A, Table 4) have reported functional roles not related to PDE4s, so far. Therefore, their association with apremilast response may contribute to the identification of novel molecules participating in apremilast’s therapeutic action.

Generally, the colocalization of association signals with cis-eQTLs strengthens the validity of the association and implies that the identified variants could be causal. In our study, more than half of the SNPs associated with the apremilast response constitute eQTLs for the respective genes in relevant-to-Ps tissues and cells. The majority of the eQTLs were identified in immune cells, thus they may modulate the inflammatory signaling of PDE4s interfering with apremilast response. It is worth mentioning the case of the intronic SNP rs35599367 in *CYP3A4* which has been functionally characterized clarifying its identification as an eQTL in the liver (Table S3). Its minor T allele has been demonstrated to alter the *CYP3A4* splicing pattern in HepG2 cells producing a nonfunctional variant that yields reduced enzyme expression [54], thus increasing drug bioavailability. In such cases, dose adjustment may be required to reduce side effects, as it has been suggested for rs35599367 and statin usage [55]. Notably, in our cohort, the T allele of this SNP is associated with a positive response to apremilast since its frequency is 11.4% in the responder group versus 1.85% in the non-responders.

Besides the identification of genetic markers, our study highlights that a higher absolute baseline PASI was associated with lower treatment response. This is in accordance with a previous pooled analysis of Phase 3 and Phase 4 clinical trials which concluded that apremilast may be particularly beneficial in more moderate disease, early in the treatment [56]. In contrast, the efficacy of apremilast treatment was not correlated with sex as has been previously suggested for systemic drugs and biological agents in Ps [57,58]. This difference in sex was partly attributed to the lower weight of women vs. men in combination with the weigh-independent standard dosing of apremilast. This men/women weight difference was also detected in our study; although, we found no difference in the weight of women in the responder and non-responder group (Table 1). Thus, the correlation of treatment response with body weight could be further investigated.

This study comprises the second pharmacogenetic study of apremilast in Ps, though in a larger number of patients compared to the first one [19], and the first association study of apremilast’s target genes. The results of our analysis should be discussed within the context of the number of samples tested; however, they offer an appreciable start towards the precision medicine goal aiming Ps. Our results have to be validated in larger cohorts of various populations. This could offer stronger associations throughout the genome to be utilized for estimating the polygenic risk score of Ps patients. Nevertheless, this integrated candidate-gene approach revealed promising biomarkers that could predict apremilast response and complies with the initiative for further investigation of the field.

## Figures and Tables

**Table 1:** Patients’ physical and clinical characteristics.

	All Patients (n = 49)	Responders (n = 22)	Non-Responders (n = 27)	*p* Value
Male/Female (M/F)	32/17	13/9	19/8	0.601
Mean baseline age (mean ± s.d.)	55.4 (±15)	54.8 (±13.2)	55.9 (±16.4)	0.954
Disease duration (years)	18.4 (±13.1)	17.1 (±10.5)	19.2 (±14.6)	0.634
Baseline weight (kg), M/F	92.8 (±19.4), 98/83.2	90.5 (±20.3), 95.9/82.4	94.7 (±18.9), 93.3/84.1	0.469
Baseline BMI	31.6 (±5.7)	32.3 (±7.3)	31.1 (±4.3)	0.513
Baseline PASI	10.3 (±6.6)	8.5 (±3.2)	11.8 (±8.3)	0.036
PASI at 6 months	6.1 (±6.4)	1.7 (±1.4)	9.8 (±6.6)	0.003
PASI at 9 months	4.7 (±4.5)	1.3 (±0.9)	7.6 (±4.2)	0.007

Note: s.d.: standard deviation.

**Table 2:** Statistically significant SNPs associated with the response to apremilast within or near PDE4 and CYP3A4 genes.

Locus	SNP	Location, HGVS Name	F_R	F_NR	MAF R + NR	MAF (EUR)	*p* Value	OR
u. PDE4A	rs12979813 (A/G)	Intergenic, chr19, NC_000019.10:g.11232027	0.4545	0.2593	0.35	0.14	4.3 × 10^−2^	0.42
	rs2305795 * (A/G)	Intergenic, chr19, NC_000019.10:g.10115376	0.5909	0.3889	0.48	0.40	4.6 × 10^−2^	0.44
d. PDE4A	rs76966440 (C/T)	Intergenic, chr19, NC_000019.10:g.10687087	0.1136	0	0.05	0.02	1.1 × 10^−2^	0
	rs892085 * (A/G)	Intergenic, chr19, NC_000019.10:g.10707416	0.6136	0.3889	0.49	0.48	2.7 × 10^−2^	0.40
	rs74942924 (G/A)	Intergenic, chr19, NC_000019.10:g.10952989	0	0.0926	0.05	0.03	3.8 × 10^−2^	NA
	rs11085752 * (A/C)	Intergenic, chr19, NC_000019.10:g.10954733	0.6136	0.3889	0.49	0.42	2.7 × 10^−2^	0.40
	rs7935 (T/C)	Intergenic, chr19, NC_000019.10:g.10994932	0.5227	0.3148	0.41	0.35	3.7 × 10^−2^	0.42
	rs12980863 (C/T)	Intergenic, chr19, NC_000019.10:g.11199195	0.2955	0.5000	0.41	0.39	4 × 10^−2^	2.39
	rs12979813 (A/G)	Intergenic, chr19, NC_000019.10:g.11232027	0.0682	0.2222	0.15	0.14	3.5 × 10^−2^	3.91
	rs420703 (C/A)	Intergenic, chr19, NC_000019.10:g.11301192	0.3409	0.5741	0.47	0.30	2.1 × 10^−2^	2.61
	rs322151 * (C/T)	Intergenic, chr19, NC_000019.10:g.11306524	0.2727	0.4815	0.39	0.23	3.5 × 10^−2^	2.48
	rs322144 (G/C)	Intergenic, chr19, NC_000019.10:g.11313027	0.3409	0.6296	0.50	0.56	4 × 10^−3^	3.29
	rs10424035 (G/A)	Intergenic, chr19, NC_000019.10:g.11400322	0	0.0926	0.05	0.01	3.8 × 10^−2^	NA
u. PDE4B	rs883824 (G/A)	Intergenic, chr1, NC_000001.11:g.64830174	0.0455	0.1852	0.12	0.18	3.6 × 10^−2^	4.77
	rs1045895 (G/A)	Intergenic, chr1, NC_000001.11:g.65432298	0.3409	0.5556	0.46	0.40	3.4 × 10^−2^	2.42
	rs1045895 (G/A) dom †		10 GA + AA/12 GG	21 GA + AA/6 GG			2 × 10^−2^	
PDE4B	rs61799396 (C/T)	Intron, chr1, NC_000001.11:g.65956761	0.3409	0.1667	0.24	0.25	4.6 × 10^−2^	0.39
	rs1937457 (C/T)	Intron, chr1, NC_000001.11:g.65981104	0.4318	0.2222	0.32	0.29	2.6 × 10^−2^	0.38
	rs12757542 (A/G)	Intron, chr1, NC_000001.11:g.66030425	0.3864	0.1667	0.27	0.28	1.4 × 10^−2^	0.32
	rs12406476 (T/C)	Intron, chr1, NC_000001.11:g.66090109	0.3409	0.5556	0.46	0.52	3.4 × 10^−2^	2.42
	rs12745871 (C/T)	Intron, chr1, NC_000001.11:g.66121693	0.6136	0.3148	0.45	0.34	3 × 10^−3^	0.29
	rs2503174 (A/G)	Intron, chr1, NC_000001.11:g.66143471	0.0909	0.2593	0.18	0.32	3.2 × 10^−2^	3.50
	rs2485381 (A/G)	Intron, chr1, NC_000001.11:g.66162049	0.0227	0.1481	0.09	0.17	3.2 × 10^−2^	7.48
	rs1890196 (T/C)	Intron, chr 1, NC_000001.11:g.66337397	0.3182	0.5741	0.46	0.48	1.1 × 10^−2^	2.89
d. PDE4B	rs11208847 (T/C)	Intergenic, chr1, NC_000001.11:g.66400358	0.0909	0.2593	0.18	0.22	3.2 × 10^−2^	3.50
	rs12118088 (A/G)	Intergenic, chr1, NC_000001.11:g.66479505	0	0.2037	0.11	0.09	1 × 10^−3^	NA
	rs765685 (G/A)	Intergenic, chr1, NC_000001.11:g.66499792	0.5455	0.3333	0.43	0.48	3.5 × 10^−2^	0.42
	rs11809759 (A/G)	Intergenic, chr1, NC_000001.11:g.66804222	0.4773	0.2778	0.37	0.41	4.2 × 10^−2^	0.42
	rs2815378 (C/T)	Intergenic, chr1, NC_000001.11:g.67044015	0.0682	0.3148	0.20	0.30	3 × 10^−3^	6.28
	rs77040148 (A/G)	Intergenic, chr1, NC_000001.11:g.67138150	0	0.1296	0.07	0.05	1.3 × 10^−2^	NA
d. PDE4C	rs62128111 (C/T)	Intergenic, chr19, NC_000019.10:g.17352405	0.1591	0.4074	0.30	0.27	7 × 10^−3^	3.63
	rs12972417 (C/T)	Intergenic, chr19, NC_000019.10:g.17414505	0.0909	0.2778	0.19	0.22	2 × 10^−2^	3.85
	rs10410487 (C/T)	Intergenic, chr19, NC_000019.10:g.17718799	0.3636	0.5741	0.48	0.43	3.8 × 10^−2^	2.36
	rs12151113 (T/C)	Intergenic, chr19, NC_000019.10:g.17748561	0.5909	0.3333	0.45	0.51	1.1 × 10^−2^	0.35
	rs2161489 (C/T)	Intergenic, chr19, NC_000019.10:g.17753643	0	0.1296	0.07	0.07	1.3 × 10^−2^	NA
	rs8108865 (C/T)	Intergenic, chr19, NC_000019.10:g.17761260	0.2500	0.0556	0.14	0.19	6 × 10^−3^	0.18
	rs4808096 (A/G)	Intergenic, chr19, NC_000019.10:g.17771371	0	0.0926	0.05	0.05	3.8 × 10^−2^	NA
	rs897753 (A/C)	Intergenic, chr19, NC_000019.10:g.18043766	0.3409	0.1481	0.23	0.20	2.5 × 10^−2^	0.34
u. PDE4C	rs4808781 (G/A)	Intergenic, chr 19, NC_000019.10:g.18306162	0.0682	0.3333	0.21	0.23	1 × 10^−3^	6.83
	rs76580597 (C/T)	Intergenic, chr 19, NC_000019.10:g.18317822	0	0.0926	0.05	0.05	3.8 × 10^−2^	NA
	rs10423674 (C/A)	Intergenic, chr 19, NC_000019.10:g.18707093	0.4773	0.2778	0.37	0.32	4.2 × 10^−2^	0.42
	rs2023878 (C/T)	Intergenic, chr 19, NC_000019.10:g.18723314	0.3182	0.1481	0.22	0.21	4.5 × 10^−2^	0.37
	rs4808907 (T/C)	Intergenic, chr 19, NC_000019.10:g.19025732	0.1818	0.4074	0.31	0.23	1.6 × 10^−2^	3.09
d. PDE4D	rs11960699 (A/C)	Intergenic, chr 5, NC_000005.10:g.58013923	0.2955	0.5185	0.42	0.40	2.6 × 10^−2^	2.57
	rs11741819 (T/C)	Intergenic, chr 5, NC_000005.10:g.58014172	0.1364	0.3333	0.24	0.23	2.4 × 10^−2^	3.17
	rs72753331 (G/A)	Intergenic, chr 5, NC_000005.10:g.58044825	0.0682	0.2222	0.15	0.07	3.5 × 10^−2^	3.91
	rs16887824 (C/T)	Intergenic, chr 5, NC_000005.10:g.58109683	0.3409	0.1111	0.21	0.18	6 × 10^−3^	0.24
	rs9283718 (G/A)	Intergenic, chr 5, NC_000005.10:g.58115743	0.3182	0.5185	0.43	0.51	4.6 × 10^−2^	2.31
	rs11742503 (A/G)	Intergenic, chr 5, NC_000005.10:g.58234354	0.3182	0.1481	0.22	0.18	4.5 × 10^−2^	0.37
	rs115791568 (A/G)	Intergenic, chr 5, NC_000005.10:g.58742066	0.1136	0	0.05	0.04	1.1 × 10^−2^	0
	rs963443 (G/A)	Intergenic, chr 5, NC_000005.10:g.58775972	0.3409	0.5926	0.48	0.41	1.3 × 10^−2^	2.81
PDE4D	rs78103527 (T/C)	Intron, chr 5, NC_000005.10:g.59381162	0	0.0926	0.05	0.02	3.8 × 10^−2^	NA
	rs697076 (C/T)	Intron, chr 5, NC_000005.10:g.59555985	0.1364	0.3519	0.26	0.23	1.5 × 10^−2^	3.44
	rs295943 (C/T)	Intron, chr 5, NC_000005.10:g.59591047	0	0.1481	0.08	0.11	8 × 10^−3^	NA
	rs177077 (C/T)	Intron, chr 5, NC_000005.10:g.59643033	0.0909	0.2778	0.19	0.24	2 × 10^−2^	3.85
	rs16890078 (T/C)	Intron, chr 5, NC_000005.10:g.59661496	0.1364	0.0185	0.07	0.08	2.4 × 10^−2^	0.12
	rs2963821 (A/C)	Intron, chr 5, NC_000005.10:g.59813075	0.3182	0.5185	0.43	0.49	4.6 × 10^−2^	2.31
d. CYP3A4	rs73159483 (G/A)	Intergenic, chr 7, NC_000007.14:g.98806461	0	0.0926	0.05	0.03	3.8 × 10^−2^	NA
	rs1203844 (T/C)	Intergenic, chr 7, NC_000007.14:g.98850871	0.5000	0.2407	0.36	0.24	8 × 10^−3^	0.32
	rs2395022 * (C/A)	Intergenic, chr 7, NC_000007.14:g.99152756	0.1136	0	0.05	0.06	1.1 × 10^−2^	0
CYP3A4	rs35599367 (C/T)	Intron, chr 7, NC_000007.14:g.99768693	0.1136	0.0185	0.06	0.05	5 × 10^−2^	0.15
u. CYP3A4	rs472660 (G/A)	Intergenic, chr 7, NC_000007.14:g.99862484	0.1591	0.0370	0.09	0.12	3.7 × 10^−2^	0.20
	rs7786877 (A/G)	Intergenic, chr 7, NC_000007.14:g.100616392	0.2273	0.4259	0.34	0.26	3.8 × 10^−2^	2.52
	rs4729597 (T/C)	Intergenic, chr 7, NC_000007.14:g.100624226	0.2727	0.5000	0.40	0.37	2.2 × 10^−2^	2.67
	rs117406702 (G/A)	Intergenic, chr 7, NC_000007.14:g.100748471	0	0.0926	0.05	0.03	3.8 × 10^−2^	NA

Note: d./u.: downstream of/upstream of; F_R: frequency of the alternative allele in responders; F_NR: frequency of the alternative allele in non-responders; NA: not applicable; OR: odds ratio; Intergenic: in respect to PDE4 or CYP3A4 genes; dom†: dominant model; * SNPs associated with autoimmune diseases (see Section 4).

**Table 3:** Statistically significant SNPs in ncRNAs associated with the response to apremilast.

Locus	SNP	Location, HGVS Name	F_R	F_NR	MAF R + NR	MAF EUR	*p* Value	OR
*ANRIL*	rs1063192 (A/G)	3′ UTR, chr9, NM_004936.4:c.*2619	0.568	0.315	0.43	0.43	1.2 × 10^−2^	0.35
*ANRIL*	rs10120688 (A/G)	Intron, chr9, NC_000009.12:g.22056500	0.636	0.407	0.51	0.50	2.4 × 10^−2^	0.39
*LINC00941*	rs12297445 (G/A)	Intron, chr12, NC_000012.12:g.30789344	0	0.093	0.05	0.09	3.8 × 10^−2^	NA
*miR4706*	rs2296316 (T/C)	Intron, chr14, NC_000014.9:g.65053528	0.591	0.333	0.45	0.46	1.1 × 10^−2^	0.35

Note: F_R: frequency of the alternative allele in responders; F_NR: frequency of the alternative allele in non-responders; NA: not applicable; OR: odds ratio.

**Table 4:** Statistically significant SNPs identified through the protein network and pathway analysis associated with the response to apremilast.

Protein (Gene) Name	SNP	Location, HGVS Name	F_R	F_NR	MAF R + NR	MAF EUR	*p* Value	OR
AKAP6 (*AKAP6*)	rs2031106 (G/A)	Intron, chr 14, NC_000014.9:g.32392503	0.182	0.056	0.11	0.12	4.9 × 10^−2^	0.26
	rs7142538 (A/G)	Intron, chr 14, NC_000014.9:g.32404235	0.114	0	0.05	0.08	1.1 × 10^−2^	0
	rs3742926 (C/T)	Missense, chr 14, NM_004274.5:c.1010	0.068	0.222	0.15	0.10	3.5 × 10^−2^	3.91
	rs7150894 (A/G)	Synonymous, chr 14, NM_004274.5:c.1155	0.523	0.296	0.40	0.34	2.3 × 10^−2^	0.38
	rs1956204 (C/T)	Intron, chr 14, NC_000014.9:g.32549256	0.136	0.315	0.23	0.20	3.8 × 10^−2^	2.91
	rs11624518 (G/A)	Intron, chr 14, NC_000014.9:g.32711145	0.205	0.389	0.31	0.29	4.9 × 10^−2^	2.48
	rs11622943 (A/G)	Intron, chr 14, NC_000014.9:g.32776891	0.159	0.352	0.27	0.39	3.2 × 10^−2^	2.87
	rs34628134 (A/G)	Intron, chr 14, NC_000014.9:g.32778457	0.455	0.241	0.34	0.23	2.6 × 10^−2^	0.38
A4 (*APP*)	rs380417 (C/T)	Intron, chr 21, NC_000021.9:g.25899847	0.023	0.185	0.11	0.22	1.1 × 10^−2^	9.77
	rs2829981 (A/G)	Intron, chr 21, NC_000021.9:g.25908384	0	0.093	0.05	0.09	3.8 × 10^−2^	NA
	rs1783024 (C/T)	Intron, chr 21, NC_000021.9:g.25966822	0.614	0.407	0.50	0.42	4.2 × 10^−2^	0.43
	rs128648 (C/T)	Intron, chr 21, NC_000021.9:g.25970679	0.136	0.315	0.23	0.28	3.8 × 10^−2^	2.91
	rs58908134 (G/T)	Intron, chr 21, NC_000021.9:g.25989938	0.068	0.222	0.15	0.18	3.5 × 10^−2^	3.91
SHAN2 (*SHANK2*)	rs10899236 (A/G)	Intron, chr 11, NC_000011.10:g.70606029	0.614	0.407	0.50	0.45	4.2 × 10^−2^	0.43
	rs12274337 (C/T)	Intron, chr 11, NC_000011.10:g.70643910	0	0.093	0.05	0.03	3.8 × 10^−2^	NA
	rs35961474 (C/T)	Intron, chr 11, NC_000011.10:g.70901955	0.136	0	0.06	0.05	5.1 × 10^−3^	0
ITSN1 (*ITSN1*)	rs2251854 (A/G)	Intron, chr 21, NC_000021.9:g.33683942	0.205	0.426	0.33	0.32	2 × 10^−2^	2.89
	rs2256797 (T/C)	Intron, chr 21, NC_000021.9:g.33785207	0.114	0.333	0.23	0.23	1.1 × 10^−2^	3.90
	rs2834269 (T/C)	Intron, chr 21, NC_000021.9:g.33828070	0.455	0.241	0.34	0.25	2.6 × 10^−2^	0.38
	rs2834287 (A/G)	3′ UTR, chr 21, NM_003024.3:c.*357	0.091	0.315	0.21	0.17	7.2 × 10^−3^	4.60
P85A (*PIK3R1*)	rs831122 (G/A)	Intron, chr 5, NC_000005.10:g.68267102	0.023	0.185	0.11	0.15	1.1 × 10^−2^	9.77
AKA12 (*AKAP12*)	rs10499266 (G/A)	Intron, chr 6, NC_000006.12:g.151251388	0.136	0.019	0.07	0.08	2.4 × 10^−2^	0.12
	rs73613423 (T/G)	Intron, chr 6, NC_000006.12:g.151263516	0.114	0	0.05	0.05	1.1 × 10^−2^	0
	rs9397389 (C/T)	Intron, chr 6, NC_000006.12:g.151308730	0.205	0.389	0.31	0.26	4.9 × 10^−2^	2.48
MEOX2 (*MEOX2*)	rs10270030 (T/C)	Intron, chr 7, NC_000007.14:g.15648375	0.318	0.148	0.22	0.35	4.5 × 10^−2^	0.37
ABL1 (*ABL1*)	rs75764711 (G/A)	Intron, chr 9, NC_000009.12:g.130794660	0	0.111	0.06	0.02	2.2 × 10^−2^	NA
ARRB1 (*ARRB1*)	rs512797 (G/A)	Intron, chr 11, NC_000011.10:g.75285037	0	0.167	0.09	0.16	4.5 × 10^−3^	NA
	rs616714 (G/T)	Intron, chr 11, NC_000011.10:g.75333596G	0.068	0.259	0.17	0.18	1.3 × 10^−2^	4.78
NKG2D (*KLRK1*)	rs2617151 (G/A)	Intron, chr 12, NC_000012.12:g.10379835	0.136	0.315	0.23	0.18	3.8 × 10^−2^	2.91
ZBTB1 (*ZBTB1*)	rs74056445 (A/G)	Intron, chr 14, NC_000014.9:g.64511474	0.023	0.148	0.09	0.09	3.2 × 10^−2^	7.48
CREBBP (*CBP*)	rs2239316 * (A/G)	Intron, chr16, NC_000016.10:g.3863994	0.091	0.259	0.18	0.25	3.2 × 10^−2^	3.5
ATF1 (*ATF1*)	rs1129406 (C/T)	Synonymous, chr 12, NM_005171.5:c.327	0.409	0.222	0.31	0.39	4.6 × 10^−2^	0.41
IκBα (*NFKBIA*)	rs696 * (G/A)	3′ UTR, chr 14, NM_020529.3:c.*126	0.568	0.352	0.45	0.39	3.2 × 10^−2^	0.41

Note: F_R: frequency of the alternative allele in responders; F_NR: frequency of the alternative allele in non-responders; NA: not applicable; OR: odds ratio; * variant associated with autoimmune disease, see “Section 4”.

## Data Availability

The original contributions presented in the study are included in the article supplementary material, further inquiries can be directed to the corresponding author.

## Supplementary Materials

The following supporting information can be downloaded at: https://www.mdpi.com/article/10.3390/genes15030369/s1, Table S1: LncRNAs that target PDE4 transcripts; Table S2: miRNAs that target PDE4 and CYP3A4 transcripts; Table S3: eQTLs associated with the response to apremilast.
