# Supplementary material for: PDE4 Gene Family Variants Are Associated with Response to Apremilast Treatment in Psoriasis
Source: Genes (Basel). 2024 Mar 17;15(3):369. doi: 10.3390/genes15030369 (PMC10970167; doi:10.3390/genes15030369)
Supplement: Supplementary file 1 [file genes-15-00369-s001.zip › Table S3.pdf]

Table S3: eQTLs associated with the response to apremilast.

Locus	SNP	P-value	Effect size*	Tissue/Cells
u. PDE4A	rs3745598	$1.5x10^{-2}$	-0.06	monocyte_LPS
d. PDE4A	rs892085	$1.3x10^{-2}$	-0.28	Tfh_memory
	rs74942924	$6.2x10^{-3}$	0.50	macrophage_IFNg
		$4.9x10^{-3}$	0.71	macrophage_naive
		$7.8x10^{-4}$	-1.95	Tfh_memory
		$2.3x10^{-2}$	-0.53	Th1-17_memory
		$4.2x10^{-2}$	-0.68	Th17_memory
		$4.5x10^{-2}$	0.21	skin
	rs11085752	$3.1x10^{-2}$	0.29	CD8_T-cell_naive
		$1.5x10^{-2}$	0.25	Tfh_memory
		$4.7x10^{-2}$	0.11	Th17_memory
	rs7935	$5.9x10^{-3}$	0.37	CD8_T-cell_naive
		$1.9x10^{-2}$	0.23	Tfh_memory
		$7.2x10^{-3}$	0.15	Th17_memory
	rs12979813	$4.7x10^{-2}$	0.04	blood
	rs420703	$1.1x10^{-2}$	0.27	Cells_EBV-transformed_lymphocytes
		$4.5x10^{-2}$	0.04	Skin_Sun_Exposed_Lower_leg
	rs322151	$2.6x10^{-2}$	0.23	Cells_EBV-transformed_lymphocytes
	rs322144	$2.9x10^{-2}$	0.36	CD8_T-cell_naive
	rs10424035	$1.8x10^{-2}$	0.78	Th1_memory
u. PDE4B	rs883824	$4.3x10^{-2}$	-0.09	CD8_T-cell_anti-CD3-CD28
PDE4B	rs61799396	$4.3x10^{-2}$	0.07	Skin_Sun_Exposed_Lower_leg
		$3.1x10^{-2}$	0.14	CD8_T-cell_naive
		$4.8x10^{-2}$	0.15	Tfh_memory
		$6.6x10^{-3}$	0.19	Th2_memory
	rs1937457	$2.6x10^{-2}$	0.06	Skin_Sun_Exposed_Lower_leg
		$4.5x10^{-2}$	0.13	Tfh_memory
		$8.1x10^{-3}$	0.16	Th2_memory
	rs12757542	$4.3x10^{-2}$	0.14	Th2_memory
		$2.5x10^{-2}$	0.08	skin
	rs12406476	$3x10^{-3}$	-0.07	Skin_Not_Sun_Exposed_Suprapubic
		$2.8x10^{-2}$	-0.06	monocyte_Pam3CSK4
		$1.3x10^{-3}$	0.10	CD8_T-cell_anti-CD3-CD28
		$2x10^{-2}$	0.33	NK-cell_naive
		$4.6x10^{-2}$	-0.08	skin
	rs12745871	$4.3x10^{-2}$	-0.04	Whole_Blood
		$5.5x10^{-3}$	-0.11	Skin
		$8.1x10^{-4}$	0.12	CD8_T-cell_anti-CD3-CD28
		$6.3x10^{-5}$	-0.11	Skin_Not_Sun_Exposed_Suprapubic
	rs2503174	$4.4x10^{-2}$	0.08	monocyte_naive
		$6.3x10^{-3}$	0.14	Th1_memory
	rs2485381	$4.8x10^{-2}$	0.12	CD8_T-cell_naive
		$3x10^{-2}$	0.30	NK-cell_naive
		$6.9x10^{-4}$	0.18	Th1_memory
	rs1890196	$5.6x10^{-3}$	0.07	monocyte_LPS

		$3.1x10^{-2}$	-0.07	monocyte_Pam3CSK4
		$3.2x10^{-2}$	-0.05	monocyte_R848
		$4x10^{-2}$	-0.09	Treg_memory
d. PDE4B	rs11208847	$4.9x10^{-2}$	-0.08	Th1-17_memory
u. PDE4C	rs10423674	$4x10^{-2}$	0.21	Cells_EBV-transformed_lymphocytes
	rs2023878	$1.2x10^{-2}$	0.35	Cells_EBV-transformed_lymphocytes
PDE4D	rs697076	$4x10^{-2}$	-0.11	LCL[#]
		$3x10^{-2}$	0.09	monocyte_LPS
		$4.5x10^{-2}$	0.09	monocyte_Pam3CSK4
		$2.2x10^{-2}$	0.19	B-cell_naive
	rs295943	$2.5x10^{-2}$	-0.16	LCL
	rs177077	$2.3x10^{-2}$	-0.18	neutrophil
		$2.3x10^{-2}$	-0.12	LCL
	rs16890078	$4.7x10^{-2}$	-0.21	neutrophil
		$5.3x10^{-3}$	-0.22	monocyte_LPS
		$4.3x10^{-3}$	-0.24	monocyte_Pam3CSK4
	rs2963821	$3.9x10^{-2}$	0.14	monocyte
		$2.6x10^{-5}$	0.25	neutrophil
CYP3A4	rs35599367	$1.9x10^{-2}$	-0.36	Liver
u. CYP3A4	rs117406702	$1.5x10^{-2}$	-0.25	Small_Intestine_Terminal_Ileum
ANRIL	rs1063192	$1.7x10^{-2}$	-0.13	monocyte_Pam3CSK4
	rs10120688	$1.6x10^{-2}$	-0.17	LCL
		$2.8x10^{-2}$	0.08	B-cell_naive
LINC00941	rs12297445	$2x10^{-5}$	-0.36	Skin_Sun_Exposed_Lower_leg
AKAP6	rs2031106	$3.3x10^{-2}$	-0.10	Skin_Not_Sun_Exposed_Suprapubic
		$3.8x10^{-2}$	-0.32	macrophage_Listeria
		$2.7x10^{-2}$	0.38	CD8_T-cell_anti-CD3-CD28
	rs11624518	$2.1x10^{-4}$	-0.65	macrophage_IFNg
		$2.2x10^{-5}$	-0.53	macrophage_naive
		$2.9x10^{-3}$	-0.36	macrophage_Salmonella
		$2x10^{-2}$	-0.35	macrophage_Listeria
		$3.3x10^{-2}$	0.30	CD8_T-cell_anti-CD3-CD28
APP	rs380417	$4.8x10^{-2}$	0.08	neutrophil
		$3.1x10^{-2}$	-0.05	CD4_T-cell_anti-CD3-CD28
	rs2829981	$4.5x10^{-2}$	-0.07	monocyte
		$2.1x10^{-2}$	-0.09	Skin_Not_Sun_Exposed_Suprapubic
		$4.0x10^{-2}$	-0.07	Whole_Blood
	rs1783024	$3.2x10^{-2}$	-0.08	neutrophil
		$1.9x10^{-3}$	-0.14	Treg_memory
		$2.0x10^{-2}$	-0.07	Treg_naive
	rs128648	$1.6x10^{-2}$	-0.06	monocyte
		$2.7x10^{-3}$	-0.12	neutrophil
		$2.9x10^{-2}$	-0.06	Skin_Not_Sun_Exposed_Suprapubic
		$2.8x10^{-2}$	-0.06	Whole_Blood
		$1.6x10^{-2}$	-0.07	monocyte_IAV
	rs58908134	$1.7x10^{-5}$	-0.11	monocyte
		$1.3x10^{-7}$	-0.23	neutrophil
		$8.0x10^{-5}$	-0.12	Whole_Blood
		$3.6x10^{-4}$	-0.06	blood
		$4.0x10^{-2}$	-0.07	monocyte_IAV
		$1.6x10^{-2}$	-0.09	Treg_naive

SHANK2	rs35961474	$3.9x10^{-2}$	0.69	LCL
ITSN1	rs2251854	$5.2x10^{-21}$	0.47	monocyte
		$4.1x10^{-12}$	-0.24	Skin_Not_Sun_Exposed_Suprapubic
		$6.1x10^{-9}$	-0.18	Skin_Sun_Exposed_Lower_leg
		$1.0x10^{-19}$	0.26	Whole_Blood
		$2.1x10^{-33}$	0.28	blood
		$5.8x10^{-3}$	0.10	monocyte_IAV
		$2.8x10^{-6}$	0.22	monocyte_LPS
		$4.8x10^{-4}$	0.14	monocyte_naive
		$3.8x10^{-5}$	0.17	monocyte_Pam3CSK4
		$5.1x10^{-4}$	0.18	monocyte_R848
		$3.6x10^{-2}$	-0.13	CD4_T-cell_anti-CD3-CD28
		$1.9x10^{-9}$	0.21	monocyte_CD16_naive
		$2.3x10^{-5}$	-0.07	skin
	rs2256797	$3.2x10^{-8}$	-0.32	monocyte
		$4.5x10^{-4}$	0.15	Skin_Not_Sun_Exposed_Suprapubic
		$2.3x10^{-3}$	0.12	Skin_Sun_Exposed_Lower_leg
		$3.7x10^{-12}$	-0.24	Whole_Blood
		$1.4x10^{-7}$	-0.15	blood
		$5.8x10^{-3}$	-0.16	monocyte_LPS
		$2.2x10^{-2}$	-0.10	monocyte_naive
		$2.4x10^{-4}$	-0.17	monocyte_Pam3CSK4
		$9.7x10^{-4}$	-0.20	monocyte_R848
		$1.7x10^{-4}$	-0.15	monocyte_CD16_naive
		$2.3x10^{-3}$	0.06	skin
	rs2834269	$9.1x10^{-3}$	0.09	Skin_Not_Sun_Exposed_Suprapubic
		$2.8x10^{-2}$	-0.23	Treg_naive
	rs2834287	$1.8x10^{-2}$	0.23	Th17_memory
		$4.5x10^{-2}$	-0.27	Th1_memory
		$4.6x10^{-2}$	0.04	skin
PIK3R1	rs831122	$2.7x10^{-2}$	-0.11	monocyte_IAV
		$4.7x10^{-2}$	-0.12	monocyte_LPS
		$4.2x10^{-2}$	-0.13	monocyte_Pam3CSK4
		$5.7x10^{-3}$	0.07	skin
AKAP12	rs10499266	$1.5x10^{-2}$	-0.70	Th2_memory
	rs9397389	$1.0x10^{-2}$	-0.37	macrophage_Salmonella
		$2.7x10^{-2}$	0.27	LCL
MEOX2	rs10270030	$2.8x10^{-2}$	-0.05	Skin_Not_Sun_Exposed_Suprapubic
ABL1	rs75764711	$1.3x10^{-2}$	-0.14	monocyte
		$4.2x10^{-3}$	-0.20	Skin_Sun_Exposed_Lower_leg
		$4.5x10^{-2}$	-0.13	Whole_Blood
		$9.1x10^{-4}$	-0.07	blood
		$3.0x10^{-2}$	-0.14	monocyte_LPS
ARRB1	rs512797	$1.2x10^{-2}$	0.14	macrophage_Listeria
		$4.4x10^{-2}$	0.11	CD4_T-cell_anti-CD3-CD28
		$6.2x10^{-3}$	0.14	CD4_T-cell_naive
		$3.7x10^{-2}$	0.17	CD8_T-cell_anti-CD3-CD28
		$1.3x10^{-2}$	0.11	CD8_T-cell_naive
		$1.8x10^{-2}$	0.12	Treg_naive
	rs616714	$4.3x10^{-4}$	-0.10	T-cell
		$3.2x10^{-2}$	0.09	Skin_Sun_Exposed_Lower_leg

		$1.4x10^{-2}$	-0.09	CD4_T-cell_naive
		$6.9x10^{-3}$	-0.11	CD8_T-cell_anti-CD3-CD28
		$3.5x10^{-3}$	-0.11	Th1-17_memory
		$6.9x10^{-3}$	-0.09	Th17_memory
KLRK1	rs2617151	$2.4x10^{-2}$	0.28	T-cell
		$3.5x10^{-2}$	0.11	Skin_Not_Sun_Exposed_Suprapubic
		$5.6x10^{-4}$	0.06	Whole_Blood
		$9.2x10^{-29}$	0.50	blood
		$4.2x10^{-2}$	-0.64	B-cell_naive
		$2.0x10^{-4}$	0.20	CD8_T-cell_anti-CD3-CD28
		$1.5x10^{-8}$	0.44	CD8_T-cell_naive
		$4.7x10^{-8}$	0.46	NK-cell_naive
		$4.1x10^{-3}$	0.19	skin
ZBTB1	rs74056445	$4.7x10^{-2}$	-0.14	macrophage_Salmonella
		$9.5x10^{-3}$	-0.07	monocyte
		$9.2x10^{-3}$	-0.12	Skin_Not_Sun_Exposed_Suprapubic
		$2.7x10^{-3}$	-0.14	Skin_Sun_Exposed_Lower_leg
CREBBP (CBP)	rs2239316	$5.1x10^{-3}$	0.03	blood
ATF1	rs1129406	$2.6x10^{-9}$	-0.14	monocyte
		$2.4x10^{-12}$	-0.27	neutrophil
		$9.5x10^{-4}$	-0.04	T-cell
		$4.1x10^{-5}$	0.09	Skin_Not_Sun_Exposed_Suprapubic
		$2.0x10^{-11}$	0.16	Skin_Sun_Exposed_Lower_leg
		$2.5x10^{-3}$	-0.08	Whole_Blood
		$1.6x10^{-4}$	-0.06	blood
		$8.0x10^{-3}$	-0.04	monocyte_IAV
		$7.3x10^{-3}$	-0.06	B-cell_naive
		$1.5x10^{-2}$	-0.05	Tfh_memory
		$3.2x10^{-3}$	0.06	skin
IKBA (NFKBIA)	rs696	$7.3x10^{-3}$	0.10	CD8_T-cell_anti-CD3-CD28

Note: d./u.: downstream of/upstream of; *: positive and negative effect sizes refer to the increase and reduction of gene expression respectively; #LCL: EBV transformed lymphoblastoid B-cell lines.
